# Evaluation of the Implementation of the NFFF Stress First Aid Intervention in Career Fire Departments: A Cluster Randomized Controlled Trial

**DOI:** 10.3390/ijerph20227067

**Published:** 2023-11-16

**Authors:** Sara A. Jahnke, Patricia Watson, Frank Leto, Nattinee Jitnarin, Christopher M. Kaipust, Brittany S. Hollerbach, Christopher K. Haddock, W. S. Carlos Poston, Richard Gist

**Affiliations:** 1 Center for Fire, Rescue & EMS Health Research, NDRI-USA, Inc., Leawood, KS 66224, USA; jahnke@ndri-usa.org (S.A.J.); jitnarin@ndri-usa.org (N.J.); kaipust@ndri-usa.org (C.M.K.); haddock@ndri-usa.org (C.K.H.); poston@ndri-usa.org (W.S.C.P.); 2 National Center for PTSD, White River Junction, VT 05009, USA; patricia.j.watson@dartmouth.edu; 3 FDNY, Counseling Services Unit, Fort Totten, NY 10314, USA; fleto343@gmail.com; 4 KCMO Fire Department, Kansas City, MO 64106, USA; richard.gist@kcmo.org

**Keywords:** clinical trials, resilience, peer support groups, emergency personnel, general trauma exposure, evidence-based intervention, firefighter

## Abstract

Firefighting is inherently dangerous, though recently concerns have shifted from traditional fireground injuries (burns and asphyxiation) to a focus on mental and behavioral health. Although firefighters are remarkably resilient, research suggests many suffer negative psychological consequences from repeated exposures to trauma. While the Stress First Aid (SFA) model has gained increased attention and adoption among fire departments as a model for behavioral health training, it has not been formally evaluated. This cluster randomized controlled trial used a crossover design comparing the immediate SFA group to delayed SFA control to test the impact of the SFA on firefighters’ mental and behavioral health changes after 10–12 months (*n* = 400; Mage = 37.6, 4.8% women). A convenience sample of 79 firefighters (Mage = 41.4; 8.7% women) provided evaluations on one or more of the training modules. Participants reported satisfaction with all training components (Peer team training 97.6%, Online SFA 94.9%, Curbside Manner 88.4%, After Action Review 89.4%) and reported success in changing personnel’s perception of their department’s ability to respond to behavioral health issues (SFA M = 3.93, Control 3.50; t = 2.52, *p* = 0.042). Future work should focus on additional resources and training to augment existing efforts to help departments continue their efforts.

## 1. Introduction

With significant declines over the past several decades in the number of fires in the United States (U.S.) nationally (e.g., only 3.8% of calls were actual fires in 2020), firefighters’ responsibilities have shifted from primarily engaging in fire suppression activities to include a broad range of emergency response operations [1]. Present-day firefighters are responsible for rescue operations, hazardous materials incidents, responding to natural disasters and domestic attacks, and providing emergency medical services [1,2]. Medical calls typically include a range of needs from simple sprains and strains to a wide variety of potentially traumatic events including a parent experiencing a heart attack, a child who has drowned in a swimming pool, a fire with possible trapped victims, or a terrorist attack [2,3]. A common saying in the fire service, as quoted by the 2005 documentary *Into the Fire*, is that “Your worst day is our everyday” [4]. Not surprisingly, it has been suggested that regular exposure to these events may have a negative psychological toll on firefighters’ mental health [5,6].

Given that repeated exposures to trauma (RET) are a necessary occupational hazard, it is not surprising that the mental health of first responders has been the focus of several previous studies [7,8,9,10,11,12,13,14,15]. Although firefighters often are remarkably resilient, research suggests that many suffer negative psychological consequences from their experiences. For instance, evidence suggests that rates of depression and depressive symptomatology among fire and emergency medical services (EMS) personnel are higher than the general population. In a population-based study of both career and volunteer firefighters from the Central United States (U.S.), researchers [16] found that approximately 12–27% (depending on the rate of substance use) of firefighters surveyed reported significant depressive symptoms ranging from moderate to severe. Tak and colleagues [13] surveyed 525 firefighters three months after Hurricane Katrina and found that more than a quarter (27%) were in the range of concern for depressive symptoms (according to the CES-D) after responding to the disaster. National U.S. data are not available about the prevalence and incidence of depressive symptoms specifically among firefighters. However, existing evidence from available studies suggests that rates of depressive symptoms among firefighters are substantially higher than the general population of the U.S., which typically are less than 19% [17].

Several studies documented high rates of alcohol use and binge drinking among firefighters, which has been hypothesized to be related to RET. Haddock and colleagues [18] found that 56% of career and 45% of volunteer firefighters in a recent population-based sample reported a binge drinking episode (five or more drinks in a sitting) during the past 30 days, which is similar to that reported by Carey and associates (58%) [19] in their sample of career firefighters in the Northeastern U.S. Both of these studies found that binge drinking rates are substantially higher than the general population nationally, which is typically under 20% [20]. North et al. [21,22] found that alcohol disorders were “endemic” among firefighters who had responded to the Oklahoma City bombing and that using alcohol as a coping mechanism resulted in poorer functioning. Research suggests that those experiencing emotional distress and post-traumatic stress disorder (PTSD) often report the development or exacerbation of risky drinking behaviors [23], which may account for some of the high rates of problem drinking among firefighters.

Many studies examining PTSD among firefighters have found relatively high prevalence rates. The rate of PTSD in the U.S. is estimated around 5% [24]. A number of studies have found rates of PTSD among firefighters to be higher than this, particularly immediately after a traumatic event [8,21,22,25]. For example, Corneil et al. [8] estimated PTSD prevalence of 22% for 203 firefighters in the U.S. from two large metropolitan departments in the Northwestern U.S. and 17% among 625 firefighters in one large metropolitan fire department in Canada. North and colleagues [22] found that 13% of firefighters responding to the Oklahoma City Bombing experienced clinically significant PTSD symptoms. Similar to the available data on depressive symptoms, national U.S. PTSD prevalence and incidence data for the fire service are not available but the data that exist are cause for significant concern.

The need for behavioral health services emerged only recently as a ubiquitous concern for fire and EMS organizations. Very little attention to the mental health impact of the job or approaches to prevention, mitigation, or treatment of negative outcomes appeared until the early 1980s [5,26]. The dominant attitude of earlier eras was described by some observers as “John Wayne” machismo, resigned to the brutality of tragic losses but convinced that real “firemen” should “tough” their way through these experiences and move on to the next [27]. The need for behavioral health assistance for fire and EMS personnel is now widely accepted in the fire service [28]. It is recognized that personnel and their families must have the resources to deal with the exposure to trauma inherent in the occupation [5]. Health and safety standards (e.g., NFPA 1500) require that employee assistance programs be made available to ensure that services are available when needed. However, research has raised serious concerns about early stress mitigation approaches commonly used with fire service personnel.

Critical Incident Stress Debriefing (CISD) was introduced to the fire service in the 1980s [29]. Proprietary workshops, often conducted in two-day seminars on the technique resulted in the formation of “CISD teams” in many emergency response organizations [30]. Prescriptions regarding the implementation of CISD became a part of training programs and industry standards. CISD appeared reasonable, responsible, and—perhaps most importantly of all—easily doable. There was, it seemed, every advantage and no particular “downside” to implementing CISD. By the mid-1990s, most fire and EMS organizations had a CISD team and, in many, participation was an expectation for personnel seeking the fast track to advancement [31].

Widespread adoption of CISD eventually led to closer scrutiny of its effectiveness [32,33,34,35]. The movement’s claims about the helpfulness of debriefing became the subject of investigation in independent research globally. Initial studies found that debriefing sessions had little effect on PTSD development. What proved most unsettling, however, was the finding that CISD had a negative impact on resilience and recovery, particularly in the best-designed studies. Despite claims from the debriefing community that these studies were flawed, intentionally biased, or otherwise irrelevant [32,33], the finding that debriefing is not effective for preventing PTSD has been consistent across nearly every controlled study published in the scientific literature [34,36]. Several reports [37,38,39], including a study of firefighters assigned to body recovery duties following a major commercial air crash [40], found that CISD inhibited recovery among subjects who received debriefing interventions. Additionally, research with firefighters has shown that when they are in emotionally intense contexts, distraction (disengaging or directing attention away from internal reactions) appears to more successfully reduce negativity and distress, and once they are in less emotionally intense contexts, if they rely only on distraction and do not also use reappraisal (i.e., fully experiencing the event’s cognitive and emotional significance) to process what has happened to them emotionally, they are more likely to have symptoms of PTSD long-term [41]. The importance of this research [41] is in pointing out that disengagement/distraction plays an important role in high emotionally intense contexts and results in reduced distress, so perhaps should not be overridden with mandatory socialization/debriefing. It also highlights that there is a danger of relying solely on distraction/disengagement, in that in the long run, if it is the only coping strategy, it could result in higher risk for PTSD [42]. As a result of findings like this, consensus reports and practice guidance documents have increasingly recommended against CISD intervention methods [43,44]. Unfortunately, in many areas of occupational behavioral health practice—and in fire and EMS organizations particularly—debriefing and related interventions are still widely used. In response, efforts to provide alternatives to CISD, such as *Psychological First Aid*, have gained momentum [45].

The 16 Life Safety Initiatives, part of the National Fallen Firefighters Foundation Everyone Goes Home program [46], were designed to create a culture around health and safety. Initiative 13 states that: “Firefighters and their families must have access to counseling and psychological support” [47]. At the second National Life Safety Summit, convened in 2007, selected researchers and practitioners were commissioned to develop white papers regarding the state of research, practice, and implementation in each initiative area, including Initiative 13. Incorporation of current knowledge regarding occupational stress exposures and evidence-based treatment became the first order of business with respect to Initiative 13 [46]. The white paper regarding Initiative 13 also broadened the scope of the initiative to include behavioral science and behavioral issues beyond the availability of counseling for personnel and their families.

While there are limited empirical data available about peer support and early stress mitigation types of interventions because of their flexible application in high stress working environments [35,48], preliminary evidence suggests positive behavioral health outcomes among military personnel with regard to post-traumatic stress symptoms and depression [49,50]. Based on existing literature from other occupational groups, expert opinion of fire service advisors, and input from experts in trauma research, it was determined that the principles of the Stress First Aid (SFA) model provided the best model for firefighter behavioral health, as called for by Initiative 13. The program was adapted from the Combat and Operational Stress First Aid (COFSA) Model of the Marine Corps [51], which was designed as a framework of actions for coworker support for ongoing stress. It includes a set of supportive actions to strengthen and enhance the application of five essential elements that have been found to mediate and possibly mitigate impacts in atypically stressful circumstances. The five elements are: (1) Promoting a psychological sense of safety; (2) Promoting calming; (3) Promoting a sense of self-efficacy; (4) Promoting connectedness (social support); and (5) Instilling hope.

**Figure 1:**
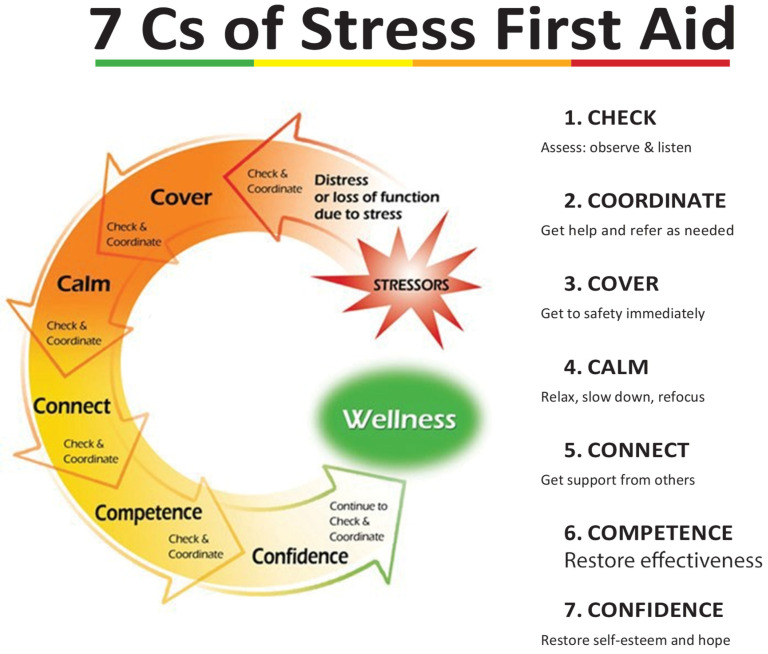
Seven Cs of Stress First Aid.

SFA added two elements to facilitate ongoing peer support and changed the names of the elements to make them easier to remember [51]. The resulting seven actions of the SFA model are: (1) Check: assess and reassess; (2) Coordinate: inform others and refer for additional care, as needed; (3) Cover: get to safety and keep safe; (4) Calm: reduce physiological and emotional arousal; (5) Connect: ensure or restore social support from peers and family; (6) Competence: restore self-efficacy and occupational and social competence; and (7) Confidence: restore self-esteem and hope. Figure 1 presents the “Seven Cs” of SFA [51] of Initiative 13 as outlined by NFFF, adapted from the COSFA manual [52].

The COSFA model [52] was based not only on “critical potentially traumatic incidents” but also on cumulative work and personal stress, as well as loss, and inner conflict/moral injury. It was not designed to specifically prevent any particular disorder, but rather to enhance individual and system capacity to weather and withstand adversity. It uses a Stress Continuum as its foundation to help reduce stigma, create a common language about stress reactions, and to help recognize when actions may be indicated, which level of intervention would be most appropriate, and how to use the SFA framework over time. Each action in the model is preceded by “Check and Coordinate” as a reminder to assess each situation and apply the action that best fits.

**Figure 2:**
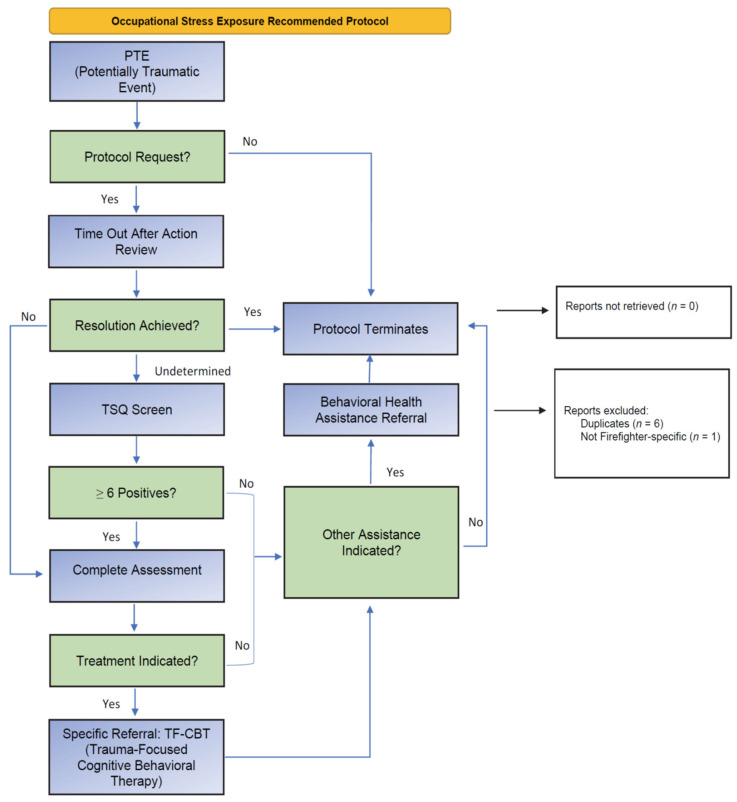
Stress First Aid (SFA) Protocol.

SFA has been adapted for fire and rescue, public safety, pretrial and probation, rail work, and healthcare settings [51]. The components of the model in all settings have been ongoing monitoring of personnel stress, wide-spread training to recognize peers or subordinates who may need additional assistance, a spectrum of one-on-one interventions, and the ability to provide personnel higher levels of care when indicated. The primary assumption of the model is that firefighters’ experiences are the result of a complex interplay of person, place, context, timing, support, personal, and public perceptions. The model also assumes that firefighters are resilient and are able to resolve many experiences without outside treatment. When individuals experience more extreme symptoms and require more focused support, they will most likely benefit from more systematic visits involving focused intervention [28]. The model notes this is best facilitated by peer-level firefighters seen as having experienced and mastered similar challenges normalizing and encouraging seeking more formal help sooner rather than later. This differs from the “peer counselor” concept common to CISD and similar approaches in that supportive contacts with coworkers are conceived as “human being 101” in high-stress work environments, rather than as interventions with a formal preventive intent. The protocol for SFA is conceived from a coworker support and leadership logic model that mitigates stress reactions both directly and indirectly via facilitation of earlier formal help-seeking, as outlined in Figure 2. The Fire/EMS SFA adaptation added self-care actions, based on input from focus group members that (a) coworker interactions are often one of their greatest sources of stress, and (b) having knowledge of self-care actions would improve peer support. SFA training for peers is a day-long course, but an awareness-level training is available online (approximately 45 min) for all department personnel.

Additional program components of the SFA logic model [47] include:

*Curbside Manner (CM)*. “Curbside Manner: Stress First Aid for the Streets” was designed as a public-facing companion to SFA for fire and rescue workers [47]. It was conceived to improve support and reduce stress in the customers first responders work with, as well as enforcing knowledge and use of the SFA model overall. Curbside Manner actions are operationalized for use with the public and organized into the **A**pproach a first responder takes, the **I**nformation they give and gather, and the **D**irection they give to the person (AID). CM actions are meant to be used as needed and as time allows, incorporated into duties in a natural, seamless way and implemented only when they do not interfere with primary duties. Training takes approximately 30 min.

*After Action Review Training*. Departments execute an “After Action Review” for their personnel, which is a systematic, guided process that formalizes the tradition of informal post-incident conversations through analyzing, refining, and improving incident response. Questions often include (1) What was our mission?; (2) What went well?; (3) What could have gone better?; (4) What might we have done differently?; (5) Who needs to know? Online training is available as well as supplemental materials to help facilitate after-action reviews such as pocket cards, flyers, and posters. This online training is approximately 30 min.

*Training for Employee Assistance Programs (EAP)*. The consensus panel determined a key need in the treatment of trauma exposure for firefighters was appropriate training of EAP personnel who receive referrals from fire departments. Thus, the “Helping Heroes” training [53] was developed, an online program specifically for EAP personnel. The training, available from NFFF and the Medical University of South Carolina (MUSC), teaches providers about the empirically validated method of intervention commonly referred to as cognitive behavioral therapy. It outlines the specific challenges first responders face when coping with trauma, as well as specific recommendations for working with this population.

While SFA has been gaining increased attention and adoption among fire departments as a model for behavioral health trainings, the program has not yet been formally evaluated. The current study was designed to evaluate the acceptability and implementation of the training components as a package.

## 2. Materials and Methods

- **Participants**

***Department Selection***. Selection of departments was based on recommendations from the project stakeholder panel and nominations from our fire service colleagues. Departments identified as possible participants were screened through an interview with project staff to determine level of motivation, resources, and commitment to program implementation. Inclusion criteria for departments included: (1) willingness to participate by department’s chief and/or his/her designee; (2) no past experience with the SFA program at the department level; (3) three or more firehouses; and (4) willingness to identify a point of contact at the department for collaboration with the research team. A total of eight departments were selected to represent a broad cross section of departments with regard to region and size. Departments were randomized to either the standard care/delayed treatment control group or the immediate intervention group using a randomization program. Each condition included: 1 large (>150 personnel), 1 medium-large (75–140 personnel), and 2 medium departments (30–74 personnel). The unit of randomization was the department since the SFA program has department-level implementation components and capitalizes on the camaraderie of firefighters. It would be difficult to implement an intervention with treatment and control participants within the same department without contamination because firefighters often spend time and live together at the station during their shift schedule.

***Participant Selection***. For departments with four or fewer stations, all firefighters on all shifts were solicited for participation as we have found that four stations are the maximum number the research team can visit in one day. For departments with more than four stations, four stations were randomly selected to participate in the study. Data collection times were coordinated for each shift at each station. The research team met with each crew, explained the study, and obtained consent from firefighters interested in being part of the evaluation. Of those solicited to participate, 98.7% agreed to participate.

- **Procedure**

**Figure 3:**
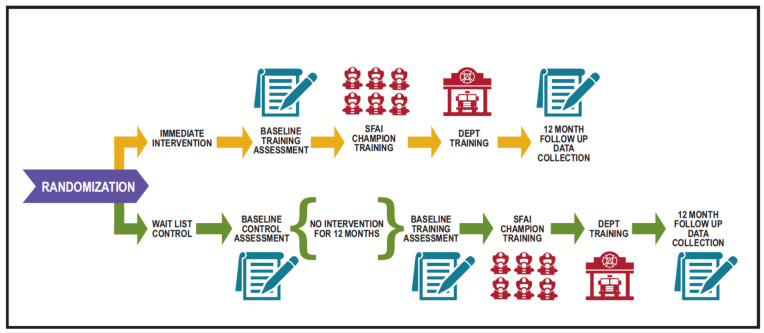
Overview of Study Design.

***Study Design***. The study was a cluster (i.e., career fire departments), randomized controlled trial (CRCT) using a crossover design comparing the immediate intervention group versus the standard care/delayed treatment control group to test the impact of the SFA program on firefighters’ 12-month mental and behavioral health changes. Four departments were randomized to each study condition. Study design and randomization procedures are detailed in Figure 3. To incentivize department participation in the control condition, participants were crossed over to the intervention arm and received the intervention after a 12-month no-intervention observation phase. The control group departments received three assessments: baseline for observation phase (Assessment 1); follow-up for observation phase and baseline for intervention phase (Assessment 2); and follow-up for intervention phase (Assessment 3). Departments randomized to the SFA condition had two assessments: baseline (Assessment 2) and follow-up for SFA phase (Assessment 3). Both the observation and SFA phases were 12 months in length. Between-groups comparisons are based on behavioral health changes from Assessment 1 to Assessment 2 for control departments and Assessment 2 to Assessment 3 for SFA departments. Within-groups changes due to intervention (both groups combined) were based on Assessments 2 and 3 for both the control and SFA department. The focus of this report is to describe the overall trial and report the main outcomes, behavioral health changes, and between-groups comparisons. Data were collected between 2015 and 2018.

The Institutional Review Board for NDRI-USA, Inc. approved the study protocol (#015-649) prior to onset of the trial, and firefighters provided consent in person before data collection began. This study has been registered with ClinicalTrials.gov (#NCT05931523).

***Implementation Procedures***. Each department enrolled in the study received training on the key SFA components. Departments in the immediate intervention group started their training protocols as soon as their department enrollment was completed. Follow-up data collection was completed 12 months later. Delayed intervention departments had the team in to solicit participation at the start of the project and continued their day-to-day operations as usual. After a year, the team re-administered the survey. At that time, the department began the program implementation process. Follow-up data collection was completed 12 months after the beginning of the intervention.

To develop the implementation plan, each department had a *planning session*. Each was visited by Fire Chief John Oates from East Hartford (CT) Fire Department. Chief Oates has been recognized for his department’s developed behavioral health program and the SFA program. Chief Oates met with the leadership and point of contact from each department to outline suggestions and ideas for program implementation including identifying personnel for the peer support team, obtaining buy-in, structure and function of the team, and lessons learned from his department’s implementation.

Each department was tasked with selecting peer-support personnel from across the department to receive the *in-person SFA* training. The training was a day-long training with Captain Frank Leto from the Fire Department of New York and Dr. Patricia Watson from the National Center for PTSD, who led the tailoring of the SFA program to the fire service culture. Typically, those attending the training became the peer team that provided resources and networking with the fire department personnel.

The SFA, CM, and AAR *online trainings*, outlined previously, were distributed to the entire department. Specific implementation varied by department based on their standard operating procedures and existing networks/communication plans within their departments, but the goal was for everyone to view and interact with the training materials.

Attempts were made to contact each department’s EAP to provide the online “*Helping Heroes*” training program. The training, provided by NFFF and MUSC [53], teaches providers about the empirically-validated method of intervention commonly referred to as cognitive behavioral therapy. It outlines the specific challenges first responders face when coping with trauma, as well as specific recommendations for working with this population.

Beyond the initial trainings, each department implemented their programs in their own ways based on the suggestions and choices from their peer-team committee. Variations included actions such as having peers meet with each crew individually, departments disseminating products with a peer team logo and contact information, inviting additional training resources into the department to discuss behavioral health challenges, and collaborating with neighboring departments to form regional teams.

- **Measures**

Outcome measures focused on social support provided by department personnel, acceptability of the training program, and perceived knowledge prior to and after the training. Due to the experiences of past research on the traditional CISD model that found iatrogenic results of program implementation, the team and stakeholders felt it was important also to evaluate behavioral health outcomes to ensure the program implementation was not causing a negative impact.

*Department Readiness to Address Behavioral Health Issues*. Questions focused on how prepared departments were to provide support for firefighters experiencing behavioral health challenges, how well-trained personnel in the department were to manage behavioral health issues, and how confident personnel were in the skills and knowledge of their company officers to handle behavioral health issues. Response options were a five-point Likert scale from “completely agree” to “completely disagree”.

*Social Support from Department*. Outcomes focused primarily on the impact of the trainings on firefighters’ feelings of being emotionally supported by personnel in their department. Questions were based on recommendations from the National Institute of Health Toolbox for Adult Social Relationship Scales [54]. Questions included: (1) I have someone who will listen to me when I need to talk; (2) I have someone to talk with when I have a bad day; (3) I have someone I trust to talk with about my feelings; and (4) I have someone with whom to share my most private worries and fears. Response options were Never, Rarely, Sometimes, Usually, and Always. Post hoc concurrent validity testing indicates the assessment questions are highly correlated with responses to both the Interpersonal Support Evaluation Checklist (*r* = 0.81, *p* < 0.001) and the Revised UCLA Loneliness Scale (*r* = 0.78, *p* < 0.001). Since the questions were intended to evaluate experiences of social support specific to the workplace, the instructions were altered to ask firefighters to indicate the amount of support he/she received from personnel at his/her department.

*Department Negative Reactions to Treatment Seeking*. The question about negative reactions to treatment seeking asked “If you sought counseling, what degree do you believe that the people in your department would…” with the list of reactions including: (1) react negatively to you; (2) see you as disturbed; (3) think bad things about you; (4) think of you less favorably; and (5) think you posed a risk to others. The questions were from the Perceptions of Stigmatization by Others for Seeking Help (PSOSH) Scale [55,56], which has been found to have strong reliability (0.80) and validity (0.72). Response options ranged from “not at all” to “a great deal” on a 5-point Likert scale and were dichotomized for the present analysis to “a lot” and “a great deal” versus not.

*Satisfaction*. Standard satisfaction questions were developed for both the in-person training for the peer team and the online trainings that were to be disseminated to the entire department. Questions focused on general satisfaction with each section of training, knowledge prior to and after the training, and how likely the firefighter thought they were to use the knowledge they gained from the training. A 5-point Likert scale was provided as response options. Those who completed the in-person training were also asked how often they had intervened with a peer in the 12 months following the training.

- **Data Analysis**

*Evaluation Plan*. The analyses in this paper are based on the post-intervention, between-groups assessment comparing departments in the SFA program to the Control condition. The SFA program is a department-level intervention; therefore, departments were randomized to conditions and firefighters in both conditions who were available after the SFA program was implemented at the intervention sites completed the assessments. The evaluation focused on factors thought to mediate the impact of the SFA program on stress-related outcomes, such as department readiness to address behavioral health issues and firefighter communication skills. In addition, we present data from convenience samples of firefighters who completed post-training feedback assessments after completing the training modules.

*Statistical Modeling Plan*. The statistical modeling plan focused on post intervention evaluations in the three assessment domains: *Department Readiness to Address Behavioral Health Issues*, *Department Supportive Communication*, and *Department Negative Reaction to Treatment Seeking*. For Department Readiness items, Likert scales were scored from 1 (Completely Disagree) to 5 (Completely Agree) and linear mixed models were used to test the mean difference and included a random effect for department. The linear mixed model includes a random intercept for each department and thus controls for the fact that firefighters are clustered within departments and for the fact that departments were the unit of randomization. Furthermore, by incorporating “department” as a random effect in our statistical models, we enhance the precision and reliability of our estimates, thereby bolstering the study’s external validity. For Department Supportive Communication, visuals present the full range of possible responses while statistical models focused on the percentage who rated the communication type of “Always” or “Usually” available. To test differences in study conditions, generalized linear mixed models were developed, which included a random effect for department. For Department Negative Reaction to Treatment Seeking, both the rates of those who rated the items as “A lot” or “A Great Deal” are presented in a visual and differences in intervention conditions were assessed with generalized linear mixed models identical in structure to those used for Department Supportive Communication. For the post-training assessments, descriptive data for satisfaction with the training and predictions for whether they would use the training along with comparisons of self-rated knowledge before and after the training were developed. Knowledge comparisons were conducted using paired *t*-tests. All analyses were conducted using the R 3.4.2 statistical programming language. We used the CONSORT reporting guidelines [57] and this trial was registered with ClinicalTrials.gov (NCT05931523).

## 3. Results

*Participant Characteristics*. Participant characteristics of those who completed the post intervention evaluation, stratified by intervention condition, are presented in Table 1. A total of 400 (60.3%) participants completed follow-up testing, out of the initial 663 participants that completed baseline assessments. The percent of females in the sample reflected national estimates for the career fire service [58]. Racial minority firefighters were overrepresented relative to the national career fire service primarily due to the large number of Asian and Native Hawaiian firefighters in the study population. Participant characteristics were similar for the two intervention groups.

**Figure 4:**
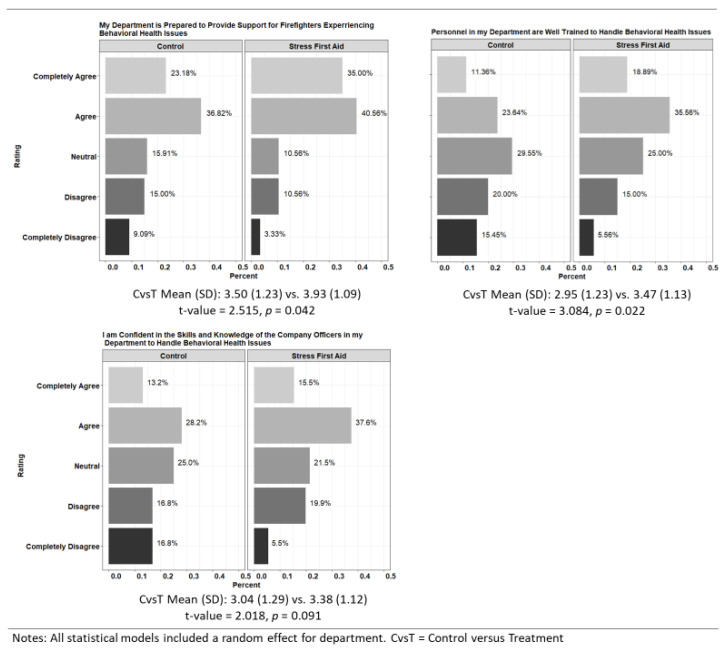
Perceptions of Department Readiness by Intervention Condition.

*Department Readiness to Address Behavioral Health Issues*. Figure 4 presents firefighter perceptions of department readiness to respond to mental health issues by intervention condition. SFA program departments rated their readiness for providing support for firefighters experiencing behavioral health issues (*p* = 0.042) and having personnel trained to handle behavioral health issues (*p* = 0.022) significantly higher than control departments. The nearly 20 percentage point difference between the two conditions in firefighters who *Strongly Agreed* and *Agreed* with the statement that personnel were well trained to handle behavioral health issues was particularly striking. Ratings for company officers having the skills and knowledge to handle behavioral health issues did not change significantly.

**Figure 5:**
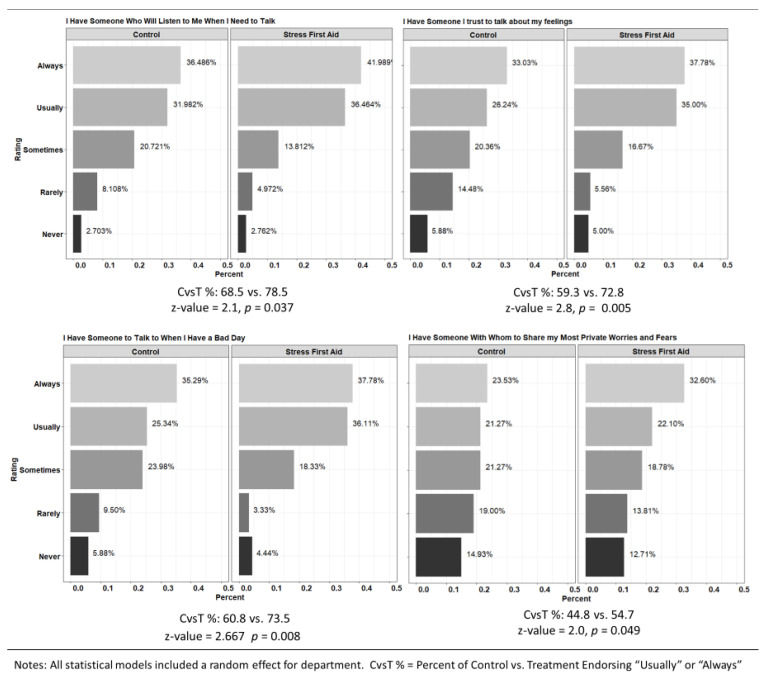
Social Support from Department by Intervention Condition.

*Social Support from Departments*. Items assessing firefighters’ perceptions of the availability of supportive communication from their department peers are presented in Figure 5. For all four types of supportive communication, firefighters from the SFA program condition consistently rated their department higher than those in Control departments. For instance, there was a 13.5-point difference favoring the SFA program departments in proportions of those who rated the item “I have someone I trust to talk to about my feelings” *Always* or *Usually*.

**Figure 6:**
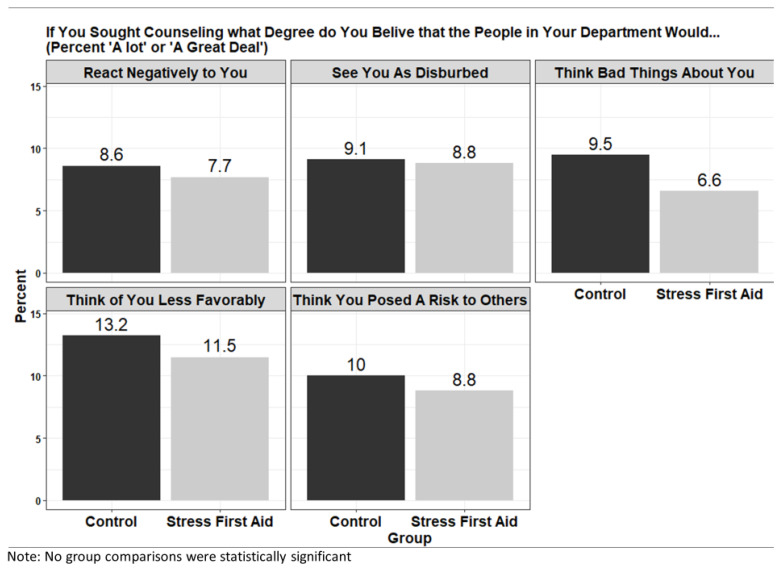
Negative Reactions to Behavioral Health Issues by Intervention Condition.

*Department Negative Reactions to Treatment Seeking*. Figure 6 presents predicted negative reactions from department peers upon learning that a firefighter sought counseling for mental health differences. While differences did not significantly differ by condition, predictions of negative reactions were unacceptably high ranging from approximately 9% to 12.3% of firefighters depending on the assessment item.

*Participant Evaluations of SFA Trainings*. A convenience sample of 79 firefighters (62.2% firefighters; average age = 41.4; 8.7% women) provided evaluations on one or more of the training modules; not all participants completed all modules of the study. Data from the participant training evaluations are presented in Table 2. All of the training modules were rated highly by the participants. Those who rated their satisfaction with the modules as “Satisfied” or “Very Satisfied” ranged from 97.6% for the Peer Team Training to 88.4% for Curbside Manner. Participants rated themselves as particularly likely to use the material presented in the Peer Team training (83.4%). Changes in self-rated knowledge of the material presented increased significantly for all training modules. The change was particularly large for the Peer Team Training ($\bar{x}=2.74$ before vs. $\bar{x}=4.14$ after).

*Training for EAP Providers*. The research team worked with department personnel to identify a point of contact with each contracted EAP. EAP leadership for the respective departments were contacted about the possibility of distributing the training to their providers who may be working with fire department personnel. One department’s EAP providers had a limited number of providers complete the training. The remaining department EAPs, despite several contacts, were unable to effectively distribute the training to their providers. Challenges cited were: (1) providers operated as contractors for the EAP and had no direct connection to the fire service; (2) for almost all EAP providers, firefighters either comprised no or a very small portion of their caseload; (3) the training was lengthy and time-intensive; and (4) providers were reluctant to change their typical practice or had no support for delivering new interventions. Also, the typical practice for an EAP is to assign service providers by location and availability. Thus, even if a provider received training, it would not alter their likelihood of providing services to a firefighter.

## 4. Discussion

In general, the SFA program trainings were well received with the majority of participants in the peer team training and taking the online trainings reporting high levels of satisfaction and increased interest in the topic. Most notably, there were statistically significant findings related to questions about how prepared the department was to provide support for firefighters experiencing behavioral health issues, how well-trained personnel within the department were to handle behavioral health issues, and how confident firefighters were in the skills and knowledge of their company officers to handle behavioral health issues. These findings indicate success in the training implementation improving the department environment related to behavioral health response.

Another noteworthy finding was the challenge the team had in accessing and engaging the EAP programs to implement trainings specific to first responders. Past research has found that EAP programs often have low rates or utilization [59]. While the concept of accessing mental health services through a standard EAP seems like a logical resource, limitations with the implementation and engagement of the systems to be responsive to the unique needs of first responders suggests relying on this as a front-line resource might have limited utility. Rather, focus should be on developing a network of resources of providers who are interested in, familiar with, and responsive to the needs of this population. Several departments in the study were successfully able to build relationships with local providers who were interested in working with the department personnel.

Changes in behavioral health outcomes over the 12-month intervention period were in the expected direction with scores in post-traumatic stress symptoms, depression, anxiety, and occupational stress decreasing and scores on resilience, coping self-efficacy, and emotional support improving, although the changes were not statistically significant. It is not surprising that changes were not larger with the short follow-up, given the observation period after program implementation was less than one year, because none of the departments were able to fully have their programs up and running right away. Typically, it took departments the first 6 months of the year to implement SFA within their department. However, the fact that the differences among these outcomes were clearly and consistently observed across domains is promising, particularly given that there is limited evidence for the effectiveness of any early supportive interventions for those who are not exhibiting significant symptoms. Given past research about the iatrogenic results of previously implemented, single-session debriefing programs [36], findings that there were trends in the positive direction related to behavioral health symptoms is promising.

As with any study, limitations to the present work exist. For instance, questions about knowledge of content areas covered within the trainings were not assessed in the year prior to implementation. Rather, perceptions of knowledge were all assessed after trainings were completed, which could have resulted in a response bias. However, results complement and mirror the longitudinally assessed domains related to perceived support within the department. There are also limitations introduced by varying participation, due to scheduling conflicts or participant attrition. While the one-year implementation was proposed for logistic and budget reasons, it became clear that the timeline for implementation of a program is significantly longer than one year. Future research should focus on long-term outcomes (e.g., changes in mental health outcomes, increases in knowledge and confidence) not only at one year, but the extended impact beyond initial training. Additionally, regular follow-up support and refresher trainings would likely improve long-term outcomes.

There are a number of personal and occupational factors that have been found to impact firefighters’ mental and behavioral health. These factors vary from person to person but may include chronic and acute traumatic experiences, physical demands, shift work, sleep disruption, cumulative stress, organizational stressors, family/home life stress, etc. [5]. Future research should further examine these factors and their impact on stress mitigation interventions. Future research should also examine the applicability of the SFA for both small career and volunteer departments [60].

Interventions designed to support the mental and behavioral health of firefighters are crucial for maintaining their wellbeing. Practical implications of this work should include efforts to further reduce the stigma associated with mental health support; additional research examining training materials designed to help firefighters recognize signs of stress, burnout, or mental health issues; increased access to mental health services; access to peer support services; coping/resilience training; and leadership support. By implementing these measures and with continued research and development, fire departments can improve the behavioral health of their firefighters and create a more supportive and resilient workforce.

## 5. Conclusions

This is the first formal evaluation of the SFA program in fire/EMS settings. Despite limitations and lessons learned for improving future research, this examination showed promising results. In conclusion, results of this study examining the acceptability, usability, and impact of the SFA program are encouraging. The program was well accepted and successfully changed personnel’s perception of their department’s ability to respond to behavioral health issues. Non-significant but positive changes in post-traumatic stress symptoms, depression, anxiety, occupational stress, resilience, coping self-efficacy, and emotional support were also noted. Future work should focus on adding additional resources and training to augment existing efforts to help departments continue their efforts.

## Figures and Tables

**Table 1:** Participant Characteristics at Post-Intervention Evaluation.

Variable	Stress First Aid	Control	All
Sample Size	180	220	400
Mean Age (SD)	38.1 (8.6)	37.2 (8.7)	37.6 (8.7)
Percent Female	5.0	4.6	4.8
Percent Race (Non-White)	22.2	19.0	20.2
Mean Years of Service (SD)	15.1 (8.4)	13.0 (8.2)	13.8
Rank in Department			
Firefighter	61.5	69.3	66.1
Officer	22.3	21.4	21.8
Chief	3.4	2.8	3.0
Other	12.8	6.5	9.1

**Table 2:** Post-Training Evaluations.

	Percent Satisfied or Very Satisfied	Percent Likely or Very Likely to Use Training	Knowledge of Material before Mean (SD)	Knowledge of Materials after Mean (SD)	Change in Knowledge *t*-Test (*p*-Value)
Training Type					
Peer Team Training (*n* = 42)	97.6	83.4	2.74 (1.01, 1–5)	4.14 (0.68, 2–5)	9.44 (<0.001)
Online Stress First Aid (*n* = 60)	94.9	66.7	2.92 (0.94, 1–5)	3.86 (0.80, 2–5)	8.11 (<0.001)
Curbside Manner (*n* = 60)	88.4	65.0	3.02 (0.93, 1–5)	3.89 (0.83, 1–5)	7.23 (<0.001)
After Action Review (*n* = 47)	89.4	63.9	3.06 (0.92, 1–5)	3.87 (0.78, 2–5)	6.77 (<0.001)

## Data Availability

Data are available upon request.
